# Selective laser sintering of distinct drug and polymer layers as a novel manufacturing strategy for individually dosed tablets

**DOI:** 10.1016/j.ijpx.2025.100338

**Published:** 2025-05-21

**Authors:** Jonas Autenrieth, Daniel Hedbom, Maria Strømme, Thomas Kipping, Jonas Lindh, Julian Quodbach

**Affiliations:** a Division of Molecular Pharmaceutics, Department of Pharmacy, Uppsala University, Uppsala Biomedical Center, P.O Box 580, SE-751 23 Uppsala, Sweden; b Division of Nanotechnology and Functional Materials, Department of Materials Science and Engineering, Uppsala University, Ångström Laboratory, Regementsvägen 1, Uppsala 751 03, Sweden; c Merck Life Science KGaA, Frankfurter Str. 250, Postcode: D033/001, DE-642 93 Darmstadt, Germany; d Department of Pharmaceutics, Utrecht Institute for Pharmaceutical Sciences, Utrecht University, Universiteitsweg 99, 3584 CG Utrecht, the Netherlands

**Keywords:** 3D-printing, Personalized medicine, Pharmaceutical technology, Individual dosing

## Abstract

Selective Laser Sintering (SLS) is an emerging additive manufacturing technology with potential for the production of personalized pharmaceuticals. In this study, we investigated a novel simplified formulation approach in SLS-based manufacturing of individually dosed, multi-layered tablets with distinct layers of pure active pharmaceutical ingredient (API) and excipient. Indomethacin (IND) was chosen as the model API, and polyvinyl alcohol (PVA) served as the excipient. Unlike conventional methods requiring powder blending, this approach utilizes separate powder tanks for IND and PVA, enabling direct printing of alternating layers in a single-step procedure.

We successfully fabricated tablets with controlled IND doses by varying the number of IND layers, maintaining consistent printing parameters across different compositions and confirming the API's chemical stability in the product. Since SLS is conventionally used for thermoplastic substances, the successful sintering of pure IND layers was a key achievement in the study, as this crystalline API is typically not printable separately. Energy dispersive X-ray spectroscopy (EDS) demonstrated the successful formation of distinct API and excipient layers. Differential scanning calorimetry (DSC) characterization revealed that the sintering process partially amorphized IND, which may enhance dissolution and bioavailability. Dissolution testing indicated that the printed tablets exhibited improved dissolution rates compared to raw IND powder.

The study successfully demonstrated the possibility of SLS-based production for personalized dosing by omitting powder blending steps. The ability to create individualized dosages with minimal excipients and simplified processing represents a step toward further investigation of SLS for clinical settings, including hospital and pharmacy-based drug production.

## Highlights

• 3D printing of adjustable drug doses for personalized medicine.
• Selective Laser Sintering (SLS) printing of distinct pure API and polymer layers.
• Simplified formulation and process development.
• Partial amorphization of API indicates improved bioavailability.

## Graphical abstract

**Figure:**
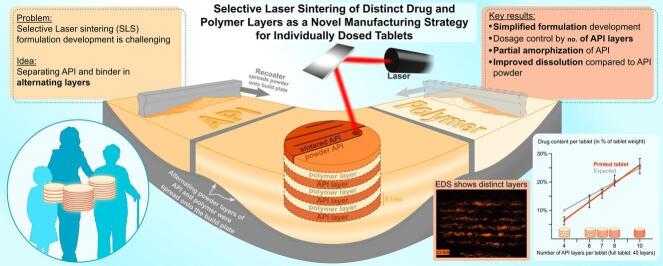

## 1 Introduction

As pharmaceutical developments advance, the discussion around personalized medicine has received more attention in recent years (Goetz and Schork, 2018). Solid oral dosage forms remain central in this context due to their various advantages, such as long-term stability, simple dosing and convenient application. (Aulton and Taylor, 2013). Traditional manufacturing methods of solid oral dosage forms, e.g., tablet compaction, are designed for mass production of drugs with standardized doses. However, special patient groups might need adjusted drug doses due to their body weight or comorbidities, especially in the pediatric or geriatric demographic (Johannesson et al., 2022). Moreover, new findings in the field of pharmacogenetics highlight the need for precision medicine for substances that can show toxicity or no effect in patients with specific genotypes (Ho et al., 2025). Currently, these patients often rely on physical manipulations of pharmaceuticals, such as breaking of tablets, as the only solution. These procedures are usually performed at home by hand or with household items. As a consequence, reproducibility is not assured and inconsistent dosing might occur (Verrue et al., 2011). The resulting imprecisions pose great risks to patients since deviations from the intended doses could lead to increased side effects or reduced drug efficacy. Although it is possible to compound medication, e.g., capsules, in pharmacies manually, this approach is time-consuming (Geersing et al., 2024) and economically inefficient due to high costs (McPherson et al., 2016).

One promising solution to close the gap between mass production and patient-individual demands lies in the emerging field of additive manufacturing (AM). This technology, commonly known as 3D printing, shows great potential for the fabrication of personalized pharmaceuticals (Trenfield et al., 2018). Within the domain of AM, Selective Laser Sintering (SLS) is of special interest. This technique uses a laser to fuse powder particles together to form a solid structure in a layer-by-layer procedure (Kruth et al., 2003). SLS printing stands out in the medical context because pharmaceutical substances often exist as powders at room temperature (Aulton and Taylor, 2013). Therefore, it is possible to print drugs directly in their raw powder form, omitting further preparation steps. This simplifies the process compared to techniques such as fused deposition modeling (FDM) or semi-solid extrusion (SSE) which require the implementation of active pharmaceutical ingredients (APIs) into filaments (Paccione et al., 2024) or gel bases (Johannesson et al., 2023), respectively. Furthermore, SLS creates fully finished prints that only require dedusting but no additional post-processing actions like drying or curing, which are needed in binder jetting (BJ) (Kreft et al., 2022) and stereolithography (SLA) (Sharma et al., 2023), respectively.

For this study, indomethacin (IND) was chosen as an API and polyvinyl alcohol (PVA) was selected as an excipient. Both substances were found to print well in SLS (Tikhomirov et al., 2023a). Previous studies that used SLS for the fabrication of individually dosed tablets were hampered by time-consuming powder preparation, as new powder blends had to be mixed for every dose (Fina et al., 2017). Although the same powder blend might be used while changing tablet dimensions for dose adjustments (Tabriz et al., 2023), this approach is limited since patients might struggle when swallowing bigger tablets (Hummler et al., 2023). In the present work, the aim was to optimize and simplify the production process by skipping the preparatory powder blending step. To achieve this, two separate powder tanks in the employed printer were used, one filled with pure API and the other with pure excipient. This novel approach allowed the printing of sandwich-like structures with alternating layers of pure API or pure polymer without prior powder mixing while maintaining consistent tablet dimensions. Dose adjustment was studied by varying the total number of API layers in the tablet. While SLS printing with two powders has been investigated by manually adding powder for each layer (Awad et al., 2019), the present approach integrated the spreading of alternating layers into the printing process, enabling a simple single-step procedure. Moreover, this study explored the printability of IND since SLS is designed for polymers and has not yet been studied for use with pure small-molecule API powders. A successful demonstration of this novel method could pose a foundation for advancing SLS toward clinical practice as a precise and adaptable manufacturing method, with higher acceptance compared to similar approaches.

## 2 Materials and methods

### 2.1 Materials

Polyvinyl alcohol (PVA, average molecular weight 45,000 g/mol, Parteck MXP® 4–88) and highly dispersed, colloidal silicon dioxide (SiO_2_, Emprove® Essential silicon dioxide) were provided by Merck Life Sciences KGaA (Darmstadt, Germany). Indometacin (IND, 98.5–100.5 % per Ph. Eur.) was provided by Sigma-Aldrich Co. (St. Louis, MO, USA). All three substances were kindly provided by Merck Life Sciences KGaA (Darmstadt, Germany). Hydrophilic PTFE filters with a pore size of 0.45 μm were purchased from VWR International AB (Stockholm, Sweden).

### 2.2 Powder preparation

The flowability of IND and PVA powder was improved by adding 1.5 % (w/w) and 0.5 % (w/w) colloidal SiO_2_, respectively. These concentrations were selected based on initial experiments which showed robust spreadability of the resulting powders and are in accordance with commonly used concentrations of SiO_2_ in pharmaceutical powders (Aulton and Taylor, 2013) as well as recommendations by the manufacturer (Evonik Industries, 2012). Each powder was sieved with a 315 μm stainless-steel sieve to remove potential powder agglomerates (VWR International AB, Stockholm, Sweden) and mixed for 15 min at 100 rpm using a Turbula T2C shaker mixer (Willy A. Bachofen AG, Basel, Switzerland). At the beginning of each day, all powders were sieved again. When PVA is labeled “fresh” in the study, it was used for printing directly as received from the manufacturer. For prints that used so-called “recycled” powder, the PVA supply tank was emptied after a finished print and the remaining, unsintered polymer was collected. This powder was then sieved and filled back into the printer. Recycled PVA had thus undergone a heating cycle at around 115–130 °C for approximately 110 min before it was sintered into a tablet structure.

### 2.3 SLS printing

**Fig. 1:**
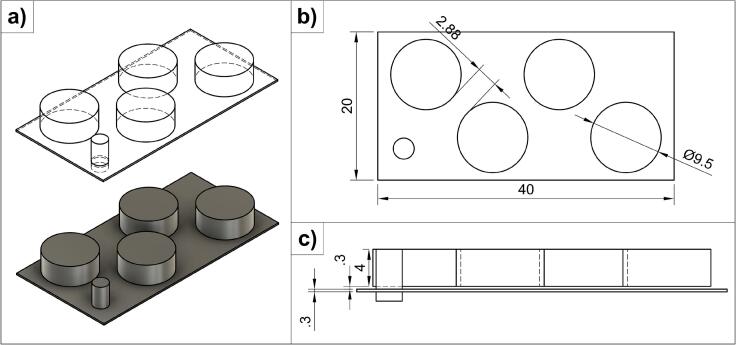
Three-dimensional model of the four printed tablets per batch, with a rectangular base and a thin column to fill empty layers (all dimensions in mm). a) isometric view b) top view c) front view.

The stereolithography (STL) model for printing (Fig. 1) was designed in Fusion 360 (version 2.0.20948, Autodesk, San Francisco, CA, USA). The STL file was then sliced in UltiMaker Cura (version 5.5.0, Ultimaker B.V., Utrecht, Netherlands) to create a geometry code (G-code) that the 3D printer can read.

The print model consisted of four cylindrical tablets with a diameter of 9.5 mm and a height of 4.0 mm. Layer height was set to 0.1 mm, which means that the tablets were 40 layers high. The tablets were placed on a rectangular base with a thickness of 0.3 mm which was located 0.3 mm beneath the tablets. A thin column with the height of the whole print was added to fill any gaps since the printer cannot process empty layers. Empty layers are layers in the STL file that do not code for any sintering, e.g., the space between the rectangular base and tablets.

Printing was conducted with a SnowWhite2 printer (Sharebot, Nibionno, Italy), which is an SLS 3D printer with a blade recoater. The printer operates with a CO_2_ laser at a wavelength of 10.6 μm. For the study, the “Reduced Area Powder Distributor” insert, provided by the manufacturer, was used to print with smaller powder volumes. PVA and IND powders were each loaded separately into one of the printer's two powder tanks. PVA was used to form warming layers during the warm-up process at the beginning of every print. During printing, the printer used powder from only one of the two tanks for each layer, alternating between the tanks. As a consequence, each layer consisted either of pure PVA or pure IND. The experimental setup is depicted in Fig. S 1.

**Fig. 2:**
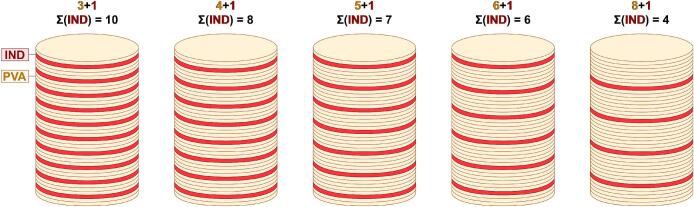
Exact order of IND and PVA layers for each tablet type. The proportions in the graphic deviate from the actual tablet dimensions for clearer demonstration.

The number of consecutive PVA layers between singular IND layers was varied. Tablets were printed using a ratio of 3 consecutive layers of PVA followed by 1 layer of IND (i.e., tablet type called “3 + 1”) as well as 4 + 1, 5 + 1, 6 + 1, and 8 + 1. Since the total layer count of each tablet was fixed to 40, the resulting number of IND layers per tablet was 10 (3 + 1), 8 (4 + 1), 7 (5 + 1), 6 (6 + 1) and 4 (8 + 1), respectively. Hence, 3 + 1 was the tablet type with the highest expected dose of IND, while 8 + 1 had the lowest dose. Moreover, all tablet types were designed in a way that the initial bottom layers, as well as the final top layer, were PVA layers. Furthermore, the STL design contained several empty layers added underneath the base to ensure that the very first IND layers from the tank were spread out without being sintered into the tablets. These first layers were thus discarded to remove possible contaminations with PVA on top of the IND tank that might occur during the filling process of the tanks or the spreading of warming layers. The exact layer sequences are depicted in Fig.2.

Print parameters were explored by conducting several test prints with different settings and layer ratios (see Table S 2). After finding print settings that allowed reproducible print success, the degradation of the API in these conditions was evaluated using NMR.

PVA layers were sintered with a laser power of 26 %, based on the maximum laser power of 14 W, and a scanning speed of 3600 mm/s. For IND layers, the laser power was 19 % and the scanning speed was set to 3000 mm/s.

The powder bed temperature during printing was set to 115 °C. After the print was finished, the tablets were left to cool down to 100 °C. They were then removed from the printer using tweezers, allowed to cool for a few more minutes at ambient conditions and carefully dedusted with pressurized air.

Each print was classified as either success or failure. A failed print was identified when obvious tablet displacement was observed or when layers delaminated during removal from the printer. Additionally, a print was declared a failure if sintered parts were discovered after sieving the excess powder that was collected in the overflow bin. This occurred when warped layers were completely dragged off the build plate by the recoater.

### 2.4 Characterization

**Fourier transform infrared spectroscopy (FTIR)** spectra were obtained using a Bruker Tensor 27 (Bruker Optic GmbH, Ettlingen, Germany) with a platinum ATR attachment, which was connected to the OPUS software (version 7.0.129, Bruker Optic GmbH, Ettlingen, Germany). Spectra were recorded in the range of 400–4000 cm^−1^ with a resolution of 4 cm^−1^.

**Nuclear magnetic resonance (NMR)** spectra were recorded using an Agilent MR-400 with OneNMR probes. Measurements were conducted at 25 °C with 16 scans, an acquisition time of 2.5559 s, a relaxation delay of 1 s, and a frequency of 400 MHz. The spectra were reported on a *δ* (ppm) scale using residual solvent signals from deuterated dimethyl sulfoxide as an internal reference for ^1^H NMR.

**Differential scanning calorimetry (DSC)** was conducted on powders using a STARe System DSC 3+ (Mettler Toledo, Columbus, OH, USA). The heating rate was set to 10 °C min^−1^, with a temperature range of 30 to 200 °C. Nitrogen was used as a purge gas, with a flow rate of 60 mL min^−1^. Measurements were normalized by sample weight.

**Thermogravimetric analysis (TGA)** was performed using a TGA/DSC 3+ instrument (Mettler Toledo, Columbus, OH, USA). Samples were heated under airflow in an uncovered aluminum oxide pan. The temperature ranged from 25 °C to 400 °C with a heating rate of 10 °C min^−1^.

**Powder X-ray diffraction (PXRD)** diffractograms were obtained using a Bruker D8 Advance (Bruker Optic GmbH, Ettlingen, Germany) with Twin-Twin optics in Bragg Brentano configuration. A Cu radiation source was used at 40 kV and 40 mA, together with a rotating sample holder and a Lynxeye XE-T detector. The scan was performed between 5 and 80 2θ (°) with a 0.021°-step size and 0.317 s dwell time.

**Friability** tests were conducted on a Pharma Test PTF1 friability tester (Pharma Test Apparatebau AG, Hainburg, Germany). For each tablet type, a total of *n* = 3 tablets was tested, choosing one tablet from three distinct batches, respectively. Following the European Pharmacopoeia (European Pharmacopoeia, 2024a), tablets were carefully dedusted with pressurized air and rotated at 25 rpm for 100 rotations. Tablets were then weighed again, and mass loss was calculated.

**Tablet mass** was determined by weighing each tablet on an XP205 DeltaRange analytical scale (d = 0.01 mg, Mettler Toledo, Columbus, OH, USA). For each tablet type, a total of *n* = 20 tablets was measured.

**Scanning electron microscopy (SEM)** images were taken with a Tescan S8000 UHR SEM (Tescan group a.s., Brno-Kohoutovice, Czech Republic) using an FEG cathode, accelerating voltage of 0.05–30 kV and magnification between 2 and 2,000,000×. The samples were sputter coated with carbon three times for 5 s at 5 A.

**Energy dispersive X-ray spectroscopy (EDS)** images were obtained with a Bruker Quantax EDS (Bruker Co., Billerica, MA, USA), using a silicon drift detector with detection of elements from ^5^B to ^95^Am and magnification of 45×. For both imaging techniques, the samples were prepared by attaching them to SEM stubs using conductive carbon pads, followed by carbon sputtering. The tablets were then halved using a razor blade, with one half prepared for cross-sectional analysis and the other for surface analysis.

### 2.5 Content determination

Content was measured on *n* = 5 tablets per tablet type, taking one tablet from five distinct batches, respectively. To determine API content, tablets were manually crushed to fine powder. A sample of around 60 mg was taken and dissolved overnight in 50 mL absolute ethanol. Next, the solution was filtered through a 0.45 μm PTFE filter and diluted by a factor of 10. Concentration was measured with a Cary 60 UV–vis-spectrophotometer (Agilent, Santa Clara, USA) at a wavelength of 319 nm, based on the absorbance maximum of the measured IND spectrum (Fig. S 2). PVA showed no absorbance at this wavelength. A calibration curve (R^2^ > 0.99) was created for concentrations of IND in ethanol between 5 and 40 μg/mL.

### 2.6 Dissolution test

The test for dissolution was conducted using a Sotax AT7 dissolution bath (Sotax AG, Aesch, Switzerland), following the standards set by the European Pharmacopoeia (European Pharmacopoeia, 2024b). Dissolution profiles (*n* = 3 per tablet type) were measured in simulated gastric fluid (SGF) at pH 1.2 without enzyme, as well as in simulated intestinal fluid at pH 6.8 without enzyme. Both buffers were prepared according to recommendations by the European Pharmacopoeia (European Pharmacopoeia, 2024c). Each release study was performed in 900 mL of buffer, kept at 37 ± 0.5 °C, and stirred at 100 rpm. Sample volumes of 3 mL were drawn at fixed time points and an equivalent amount of fresh buffer was added to the dissolution vessel. After filtering with a 0.45 μm PTFE filter, absorbance was measured with a Cary 60 UV–vis-spectrophotometer. Calibration curves for both buffers (R^2^ > 0.99) were created with IND concentrations between 5 and 40 μg/mL.

### 2.7 Statistical analysis

The statistical evaluation of the print success rates was conducted in R (version 4.2.3, R Foundation for Statistical Computing, Vienna, Austria). A generalized linear model was used with a binomial error and a logit link function due to the binomial nature of the response variable, which is either “success” or “fail”.

## 3 Results

### 3.1 Printing performance

**Fig. 3:**
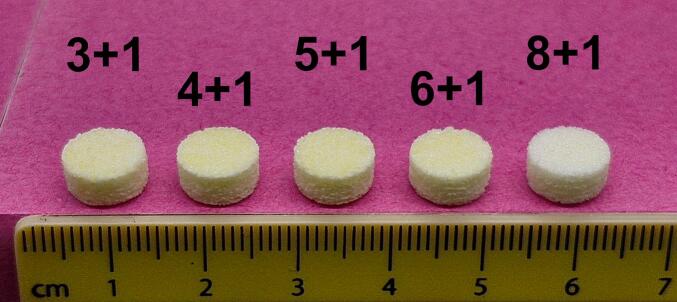
One printed tablet of each tablet type lined up next to each other.

FTIR analysis was conducted on IND powder to confirm that the API absorbs light in the wavelength of the laser used (see Fig. S 3). All layer ratios were successfully printed into tablets with consistent dimensions (Fig.3). The powder bed temperature was set to 115 °C, leading to fluctuations of the environmental temperature in the print chamber between 125 °C and 135 °C during printing. This ensured that the temperature was below the melting points of IND (158–162 °C) (European Pharmacopoeia, 2024d) and PVA (160–240 °C) (Merck KGaA, 2025) to prevent melting in the powder supply tanks. NMR analysis of a 4 + 1 tablet confirmed that the API did not undergo any degradation during laser exposure and remained intact in the printed tablet (Fig. S 4.1-S 4.3). A series of experiments was conducted to determine suitable printing parameters for the powders used (see Table S 2). The most common cause for print failure was warping of the layer, which caused the recoater to collide with the uneven tablet surface. It was found that the initial tablet layers were prone to warping when printed directly onto the powder bed. Consequently, the rectangular base at the bottom of the print model was added as a heat tank to facilitate heat distribution across the powder surface. Furthermore, it was observed that tablets had high failure rates if the bottom or top layers consisted of IND. Those IND layers tended to delaminate if they were not enclosed by PVA layers from both sides. To ensure structural integrity, all tablet models were designed to use PVA for both the first and last layer.

**Fig. 4:**
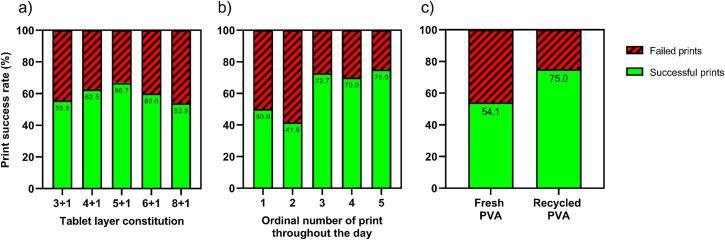
Rate of successful prints and failed prints as a function of a) ratio of PVA layers to IND layers b) number of print throughout the day c) type of PVA used. Sample sizes are summarized in Table S 1.

Due to the varying layer compositions across different tablet types, it was observed that all tablet types showed their respective best print performance with slightly different printing settings. Nevertheless, it was decided to print all tablet types with the same parameters in this study, using the most suitable parameters for 5 + 1 tablets for all tablet types since it is the medium dose. This ensured that only the number of IND layers had to be changed for the production of different doses, enabling comparability and making it easier to implement this technique into clinical practice. Despite this advantage, the use of consistent parameters resulted in a compromise. As expected, 5 + 1 tablets demonstrated the greatest print success rate since they were the standard for the set parameters, while both higher and lower doses showed an increase in print failures (Fig.4).

**Fig. 5:**
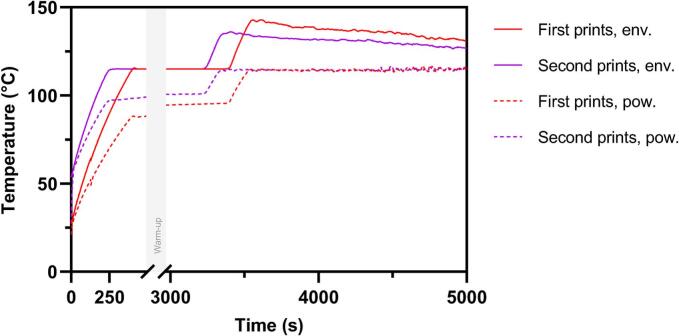
Temperature log during printing of powder bed (pow.) and environmental air (env.) in the print chamber. All temperature logs of the respective prints were averaged. The number of prints conducted per ordinal number is mentioned in Table S 1.

Moreover, the success rate of prints appeared to vary depending on the ordinal number of the print throughout the day. As the day progressed, the reliability of the printer seemed to increase. This trend can be attributed to the instrument's warm-up process. Since all prints in a day were conducted in immediate succession, the internal parts of the printer would start at room temperature for the first print but would already be heated up for the following prints. When analyzing the temperature log of the printing process (Fig.5), this change in starting temperature becomes evident. It was observed that the temperature gradients of the print bed and environment behave very differently during the first print compared to other prints on the same day. In the graph, the temperature of the powder bed and environmental air of the first prints in a day are compared to the second prints in a day. Here, the second prints are used as a representative for all prints after the first ones (second, third, fourth, and fifth) because the temperature graphs are almost identical for all of these (see Fig. S 5.1-S 5.2). Despite this, no improvement of success rate was observed for the second print of the day. The reason for this exception remains unclear. More prints could be run to determine if the low success rate persists before making final conclusions.

**Fig. 6:**
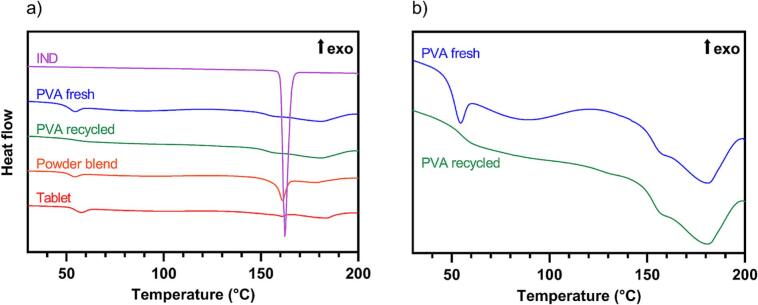
a) Normalized DSC thermograms showing all materials. b) Enlarged comparison of the DSC thermograms of fresh and recycled PVA.

Finally, it was found that there was a difference in print success rate between fresh and recycled PVA. The aged and recycled PVA exhibited better sintering properties than fresh PVA. As depicted in Fig.6, DSC analysis confirmed that PVA is a semi-crystalline powder that shows different thermophysical properties depending on whether it is fresh or recycled. The fresh PVA sample shows a clear peak at around 50 °C, which is reduced in the recycled sample. This peak is most likely attributable to enthalpy relaxation which might have occurred during storage of the powder (Menczel et al., 2009). In contrast, the recycled powder likely experienced sufficient relaxation during printing, undergoing structural rearrangement and eliminating this phenomenon. Similar heat-induced modifications of PVA have previously been described (Bershtein et al., 2012). The differing DSC curves indicate that the aging process of the powder has a relevant effect on its printing behavior when it experiences thermal stress in SLS. This suggests that the recycling of PVA may improve print consistency. Further investigations using TGA ensured that the fresh PVA is mainly water-free with a water content of less than 2 %, which means that the observed aging of preheated PVA is not simply a drying process (Fig. S 6). Since the aging of the powder was not the primary focus of this study, the phenomenon was not explored in greater detail. Further studies should be conducted investigating the underlying causes of the improved printability of recycled powder compared to fresh powder. If the improved printability of recycled powder can be confirmed, pre-heating of the polymer could be implemented into the process to make the method more reliable.

It should be noted that statistical analysis resulted in no significant difference in success rates regarding tablet type, number throughout the day, or PVA type. However, the trend in the abovementioned observations should be investigated further to clarify how the statistical analysis performs in a larger sample size. A more extensive dataset may help validate the observed trends and identify potential influencing factors. The number of prints for each analysis can be found in Table S 1.

**Fig. 7:**
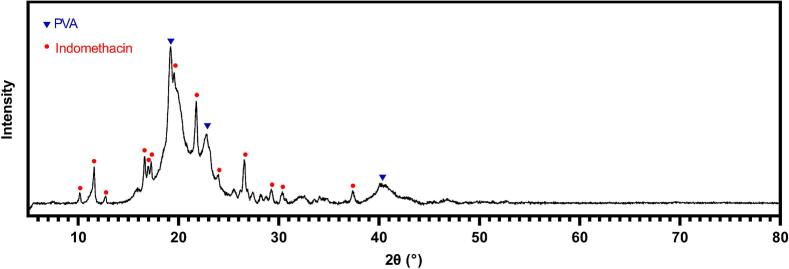
PXRD analysis of a crushed tablet. Peaks were assigned to PVA and IND diffractograms, and marked with the respective symbol of the substance that caused the peak in the mixture.

In addition to the properties of PVA, DSC analysis also shows notable differences regarding IND before and after sintering. As seen in Fig.6, pure IND powder exhibits a large melting peak around 160 °C which underlines the crystallinity of the substance. When mixing fresh PVA and IND in a ratio of 3 + 1, the unsintered powder blend still shows a smaller, clear melting peak of IND. However, in a crushed 3 + 1 tablet, the melting peak is barely visible anymore. This indicates possible amorphization of the IND during the sintering process. The melting of the substances during printing could lead to the formation of an amorphous solid dispersion at the interface between IND and PVA layers in which the polymer sterically hinders the recrystallization of the small molecule API, as observed in hot-melt extrusion (Bhujbal et al., 2021). Moreover, IND is a glass forming ability class III drug, which indicates its high stability in the amorphous state (Blaabjerg et al., 2019). To investigate the crystallinity further, the crushed 3 + 1 tablet was analyzed using PXRD (Fig.7). The spectrum was compared to a spectrum of pure PVA (data not shown) and a reference diffractogram of IND (Cox and Manson, 2003). The diffractogram of the tablet shows all peaks from both the PVA and IND spectra. This indicates that the crystallinity of raw IND is maintained to a certain extent in the printed form, even though this does not exclude crystallographic changes.

### 3.2 Friability

**Fig. 8:**
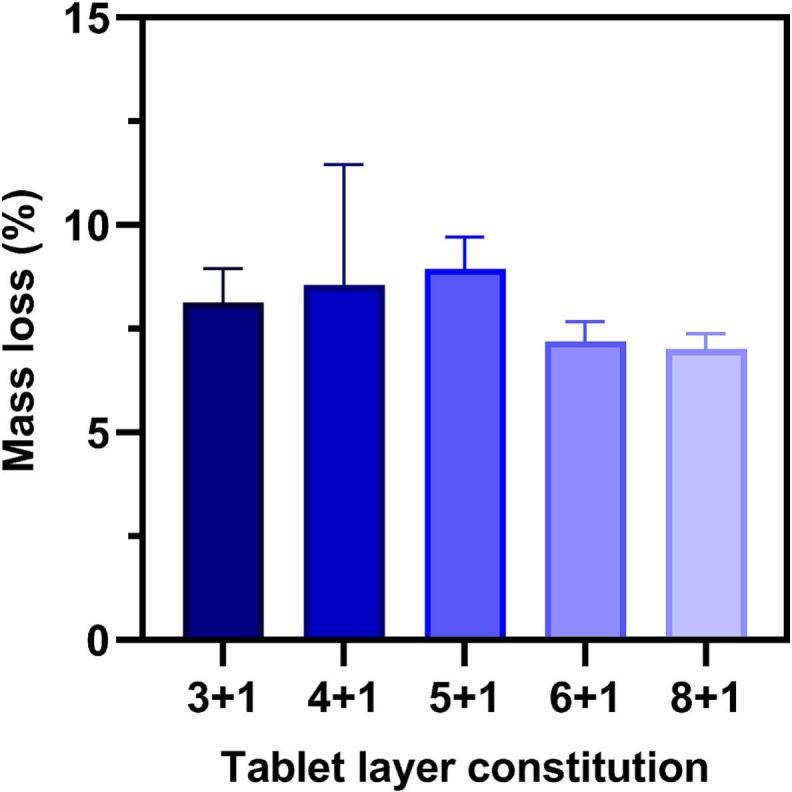
Friability analysis of all tablet types (*n* = 3 per type). Results are depicted as average ± standard deviation.

The average friability for all tablet types was found to be between 7 and 9 % (Fig.8). No tablet broke during testing and there was no visible correlation between friability and IND dose. The measured values were higher than friability in other studies on sintered tablets (Fina et al., 2017; Tikhomirov et al., 2023a). Although the measured friability was higher than the limit of 1 % set by the European Pharmacopoeia (European Pharmacopoeia, 2024a), the tablets remained structurally stable, showing sufficient robustness for further handling and analyses. Therefore, the tablets' mechanical stability is unlikely to present any problems from a future clinical perspective. If SLS is used on-site for production in hospitals or pharmacies, the tablets will not experience the same level of mechanical stress as conventional pharmaceutics which often undergo extensive handling and transportation before reaching the patient. Thus, the higher friability may not impact their practical application in controlled dispensing environments.

In this study, the test for friability was limited by small batch sizes and deviated from instructions by the Pharmacopoeia. Therefore, the results only give an indication and follow-up studies with larger batches are needed for proper comparisons.

### 3.3 Tablet mass

**Fig. 9:**
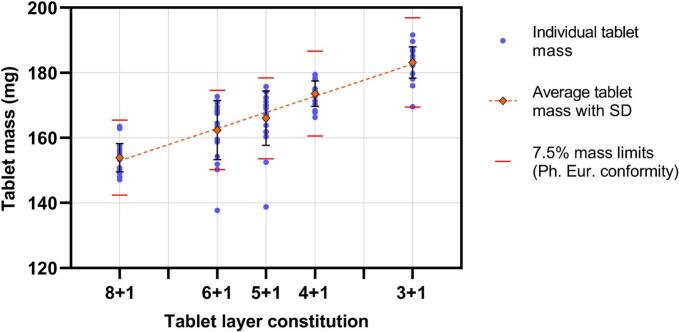
Tablet masses with individual weights, averages, and tolerance limits according to the European Pharmacopoeia.

The measured average tablet mass exhibited a linear trend across different tablet compositions (Fig.9). With a higher dose of IND, the mass increased, which aligns well with previous studies that correlated API content with higher tablet mass (Tikhomirov et al., 2023a). The individual values for mass were mostly within the limits of 7.5 % set by the European Pharmacopoeia in the test for uniformity of mass of single-dose preparations (European Pharmacopoeia, 2024e). A total of 3 tablets among all prints did not comply with these limits. Interestingly, all of those outliers were tablets that were located on the outer left border of the print plate. This leads to the conclusion that the deviation is induced by a mechanical defect in the printer that causes inconsistent powder bed temperature. This theory is supported by imaging studies that have shown variances in temperature distribution across the build plate in SLS printers (Tikhomirov et al., 2023b). A less likely assumption could be varying laser power at this position. Tablets printed in this position were therefore not used for further measurements.

### 3.4 Drug content

**Fig. 10:**
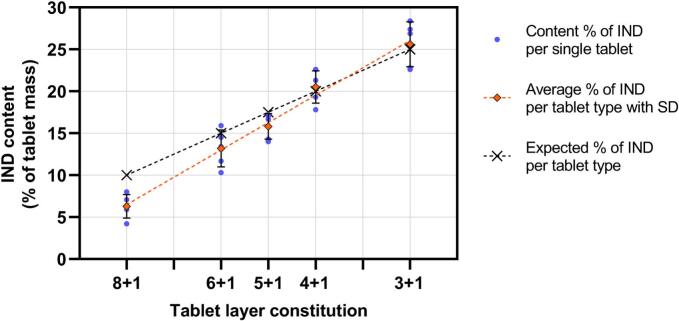
IND content in tablets as a percentage of the tablet weight with individual measurements, averages, and the expected content depending on the ratio of IND layers to total layers.

By adjusting the number of IND layers per tablet, the dose was set as a percentage of the tablet weight. The material composition of tablets depending on their type is summarized in Table1. Fig.10 shows the number of IND layers, the calculated weight share of API, and the measured weight share. There is a clear linear correlation between the weight share of IND in tablets and the number of IND layers. For higher doses, the results are nearly identical to the expected values. In lower doses, the measured values deviate further from the expected API content.

**Table 1:** The ratio of IND layers to total layers for each tablet type, the IND content expected from this ratio, and the measured content (*n* = 5 per type).

Tablet type	Number of IND layers / total layers per tablet	Expected IND content in tablet (%)	Measured IND content in tablet (%)
3 + 1	10/40	25.0	25.6
4 + 1	8/40	20.0	20.5
5 + 1	7/40	17.5	15.8
6 + 1	6/40	15.0	13.2
8 + 1	4/40	10.0	6.3

**Fig. 11:**
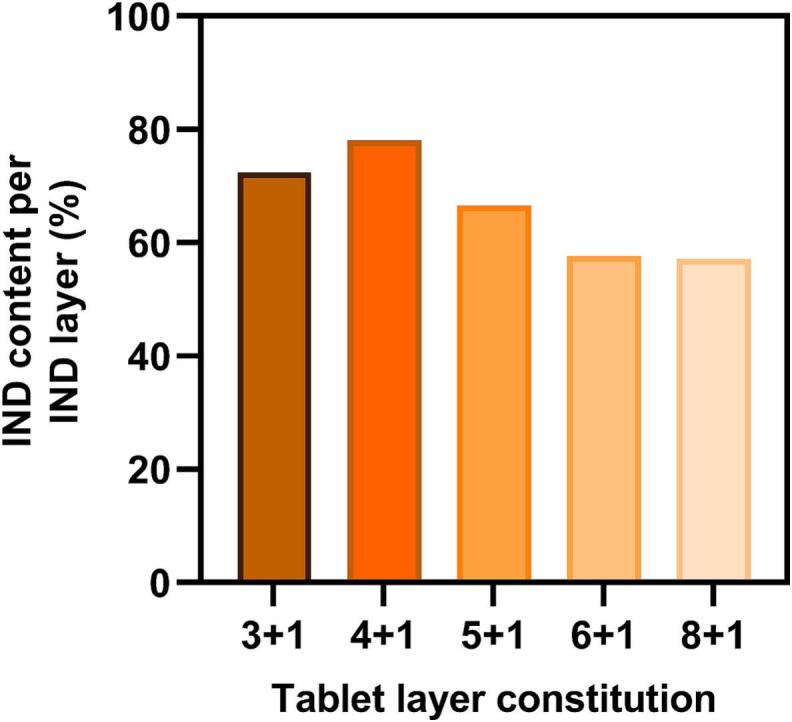
IND content in the collected powder which would be spread out as the first pure IND layer in the tablets (*n* = 1 per type).

One possible explanation for the low IND content in 6 + 1 and 8 + 1 tablets might be cross-contamination that occurs between the powder tanks during printing since the recoater moves across both powder tanks for each layer (see Fig. S 1). To investigate this hypothesis, a print was started for each tablet type. The printing process was paused right before the powder for the first IND layer in a tablet would be applied onto the build plate. All powder currently located on the build plate was removed and an aluminum weighing boat was placed on the plate. The print was then continued to allow the recoater to spread out the topmost layer of the IND tank. Due to the previous intervention, this powder fell into the weighing boat and could be collected. Content measurements of the unsintered IND layer revealed that the amount of API gradually decreases when more consecutive layers of PVA are spread, with a reduction down to approximately 60 % IND content for 8 + 1 tablets. (Fig.11). This suggests that PVA particles remain on the surface of the IND tank when the powder is spread as a new print layer as excess powder is dragged over the other tank before it reaches the overflow bin. Although this explanation matches the content measurements of 8 + 1 tablets, which contain around 60 % of the expected IND dose, it is not consistent with higher-dosed tablets. For example, 3 + 1 tablets still show cross-contamination in their IND layers even though their drug content is very close to the expected value. One possible factor could be the slightly varying bulk densities of the powders, which cancel out the contamination effect in some tablet types. Another hypothesis could relate to the fact that cross-contamination occurs in both powder tanks since IND is also being dragged over the PVA tank when spread out. Hence, both cross-contaminations may partially neutralize each other as the number of IND layers in the tablet increases. The phenomenon of cross-contamination could be avoided by modifying the printer setup. Further studies may investigate the design of a printer equipped with two powder tanks that are positioned at a 90° angle to each other, each paired with its own recoater. This way, alternating layers could be spread from the side and the top, respectively, avoiding contact with the other tank.

**Fig. 12:**
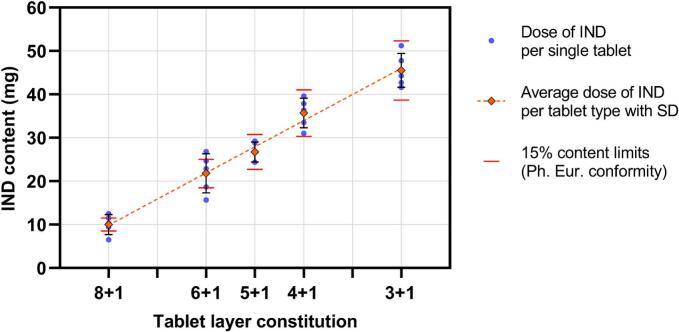
IND dose per tablet in mg, with individual contents, averages and tolerance limits according to the European Pharmacopoeia.

When analyzing the absolute IND doses in tablets instead of weight share, the limits for content uniformity by the European Pharmacopoeia (Test A) (European Pharmacopoeia, 2024f) can be calculated. For higher doses, all samples fell within the uniformity limits (Fig.12). In 6 + 1 and 8 + 1 tablets, some of the measured doses deviate by more than 15 % from the average. Due to the low doses, the limits are narrow and the variation that arises during printing appears to be too extensive. This suggests that dose precision may be challenging at lower concentrations, potentially requiring process modifications to improve uniformity. Moreover, the small batch sizes in this study only allowed for testing content uniformity between batches, but not within batches. To make definite statements on compliance with criteria set by the European Pharmacopoeia, follow-up studies with larger batch sizes are required.

### 3.5 Scanning electron microscopy (SEM) and energy dispersive X-ray spectroscopy (EDS)

**Fig. 13:**
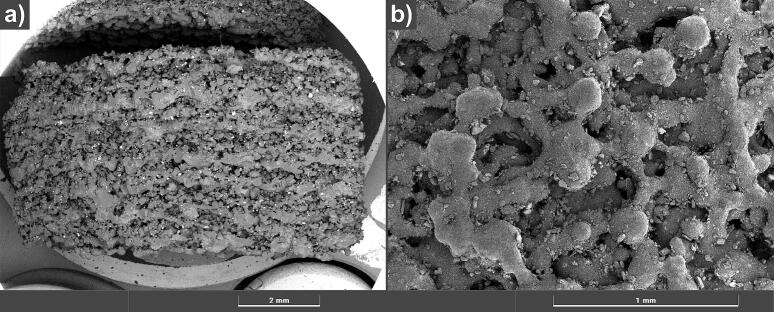
BSE SEM images showing the inside of a halved 5 + 1 tablet. The measuring parameters are a) magnification = 20.0, HV = 5 keV b) magnification = 60.6, E_0_ = 10 keV.

SEM images, presented in Fig. 13, show a highly porous and granular structure of the tablets, which is commonly found in SLS printing (Trenfield et al., 2023). Individual powder particles are visible as spherical morphologies which form bridges between each other. This demonstrates the successful sintering process. No clear structural differences can be observed between different tablet types. Drug-containing layers appear brighter in the image which can be attributed to the higher atomic weight of chloride in IND, indicating its presence in these areas. However, complete separation of layers is not possible since they appear to have agglomerated or fused into each other. This suggests a gradual composition change between layers which might affect drug release characteristics.

**Fig. 14:**
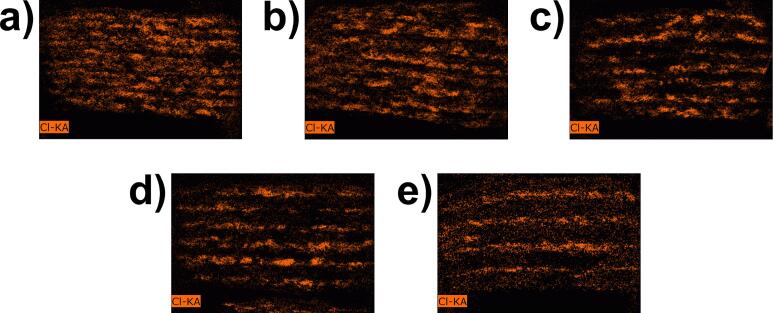
EDS pictures showing the inside of halved tablets with elemental mapping of chlorine (orange), 45× magnification. The depicted tablet types are a) 3 + 1 b) 4 + 1 c) 5 + 1 d) 6 + 1 e) 8 + 1.

EDS analysis found that the tablets mainly consist of carbon and oxygen in combination with a low concentration of silica. This is in accordance with the elemental structure of the excipients used, PVA and SiO_2_. As depicted in Fig.14, isolated spots of chlorine indicate the presence of IND which is limited to the position of singular layers. The observation proves the successful implementation of the sandwich process, leading to mostly separated PVA and IND layers, respectively. Moreover, the measured atomic percentage of chlorine steadily decreases along different tablet types, retrieving 4.44 % for 3 + 1, 3.49 % for 4 + 1, 2.32 % for 5 + 1, 1.82 % for 6 + 1, and 0.49 % for 8 + 1. Although these values cannot be used to determine the absolute IND content per tablet, they reflect the decreasing dose of IND among the samples, correlating well with the drug content depicted in Fig.10. Apart from the bright IND layers in the images, lower chlorine signals are found within the whole tablet. This could be the result of a possible contamination between the layers or a contamination that occurred during tablet sectioning for analysis. However, this signal is very evenly distributed, which raises the suspicion that it is only noise.

### 3.6 Dissolution profile

**Fig. 15:**
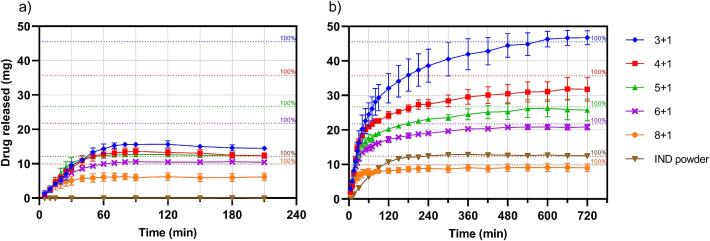
Dissolution profiles of printed tablets with their respective IND content marked as 100 % in the graph. Measurements conducted in a) pH 1.2 b) pH 6.8.

First, dissolution was tested at pH 1.2 to simulate the gastric environment (Fig.15a). All tablet types exhibited an incomplete drug release. This can be attributed to the fact that IND is a carboxylic acid and hence shows low solubility in acidic environment. Depending on the tablet type, different levels of supersaturation were reached before the concentrations of dissolved IND stabilized at similar values. Interestingly, the unsintered IND powder used as a reference showed virtually no dissolution at all. This suggests that the sintered tablet matrix facilitates the dissolution of the insoluble drug, thus enhancing the availability of IND in gastric conditions. In a physiological context, this could lead to an improved bioavailability.

During dissolution, the time required to reach maximum supersaturation mostly corresponded to the average time of full tablet disintegration, which is displayed in Table2. There is a clear trend of faster disintegration in tablets with lower IND content. This might be due to swelling of the PVA, which means that tablets with more PVA absorb water faster. These results align well with previous studies that correlated higher API content with slower disintegration in SLS printed tablets (Fina et al., 2017).

**Table 2:** Average time ± standard deviation for when full disintegration of tablets was observed during dissolution tests.

Tablet type	Disintegration time in pH 1.2 (min)	Disintegration time in pH 6.8 (min)
3 + 1	73.3 ± 11.5	86.7 ± 30.6
4 + 1	51.7 ± 23.6	86.7 ± 5.8
5 + 1	53.3 ± 11.5	48.3 ± 36.2
6 + 1	56.7 ± 5.8	66.7 ± 20.8
8 + 1	38.3 ± 12.6	36.7 ± 20.8

Another measurement regarding intestinal release was conducted at pH 6.8 (Fig.15b). Initially, all tablets exhibited a quick-release profile. However, for higher IND doses, this initial release is incomplete. After disintegration of the tablets, the dissolution profile switches to a slow release that reaches a plateau after several hours. Similar dissolution profiles of sintered tablets have been described in the literature (Kulinowski et al., 2021). A possible explanation could be that the sintered tablet disintegrates into small aggregates which are relatively stable. The remaining API that was not released from disintegration might be trapped inside these bigger particles. Consequently, it requires more time to diffuse out.

This hypothesis is reinforced by the raw IND powder control sample which also showed fast dissolution, but without the prolonged release profile in the second half. In contrast to the measurement in pH 1.2, all tablet types reached a total release of around 100 %. Since the raw IND powder also reaches 100 % dissolution, it is not possible to make statements on the effect of the sintered matrix on the API's solubility at higher pH. However, when comparing dissolution speed, the tablets demonstrated much faster initial release than the reference powder. This implies that the sintered tablet structure accelerates the dissolution process, which may enhance drug absorption and therapeutic onset.

## 4 Conclusion

In this study, we successfully 3D-printed individually dosed tablets using SLS. Our novel approach enabled the direct printing of distinct API and excipient layers, eliminating the need for powder blending and streamlining the production process. Precise dose adjustments for higher doses were conducted by altering the total number of IND layers in the print. The resulting API weight share in each tablet directly correlated with the ratio of IND layers to total layers. Tablets with low doses exhibited dose inaccuracies, which should be optimized in follow-up studies by adjusting printing parameters. Moreover, cross-contamination could be avoided by modifying the machine setup to position the powder tanks at a 90° angle to each other.

The printed tablets exhibited favorable dissolution properties compared to raw IND powder, which is currently used in manually filled capsules for dose adjustments. Partial amorphization of the crystalline API during the printing process suggests potential for increased bioavailability and faster therapeutic onset.

A key achievement of this study was the successful sintering of pure IND layers, despite SLS being conventionally used for thermoplastic polymers. Although previous studies have printed powders with high concentrations of other crystalline APIs (Kulinowski et al., 2021), IND is typically not printable. Our novel approach stabilized a single IND layer within a polymer sandwich structure, enabling the use of pure IND powder for printing. This innovation paves the way for exploring SLS printing for a broader range of pharmaceutical substances with different chemical structures. Moreover, the consistent printing parameters across various tablet types underscore the potential for reliable, scalable manufacturing. The compact size and ease of operation of the SLS printer make it a promising candidate for integration into clinical settings such as hospitals and pharmacies.

The minimal requirement for excipients can be beneficial for patients with allergies. In conventional tablet compaction, many excipients are needed to realize the production process (Aulton and Taylor, 2013). In the present work, however, only two excipients had to be combined with the API to produce tablets with adequate mechanical strength and release properties. Furthermore, this technology could be explored for veterinary applications where excipient sensitivities vary across animal species (Thomazini et al., 2024).

A limitation of this study was the production of small batch sizes. The limited number of tablets available for measurements required downscaling of some tests set by the European Pharmacopoeia. While this proof-of-concept study offered valuable insights into the properties of the printed tablets, follow-up studies with larger batch sizes are needed to carry out comprehensive assessments of content uniformity and friability, and to make definite statements on whether the tablets fully comply with the criteria in the Pharmacopoeia.

Further research should focus on further optimizing dose fine-tuning. While adjustments in the number of IND layers allow incremental changes in dosage, additional strategies such as modifying tablet dimensions could enhance precision. Addressing potential cross-contamination between powder tanks and improving content uniformity in lower-dose formulations will be critical for clinical adoption. Overall, our findings support the potential of this novel approach in SLS as a flexible and efficient tool for personalized medicine, adding to ongoing advancements in patient-specific drug manufacturing on demand.

## CRediT authorship contribution statement

**Jonas Autenrieth:** Writing – original draft, Visualization, Validation, Methodology, Investigation, Conceptualization. **Daniel Hedbom:** Writing – review & editing, Investigation. **Maria Strømme:** Writing – review & editing, Supervision, Funding acquisition. **Thomas Kipping:** Writing – review & editing, Conceptualization. **Jonas Lindh:** Writing – review & editing, Supervision, Funding acquisition, Conceptualization. **Julian Quodbach:** Writing – review & editing, Conceptualization.

## Funding

This work is conducted within the Additive Manufacturing for the Life Sciences Competence Center (AM4Life). The authors gratefully acknowledge financial support from Sweden's Innovation Agency VINNOVA (Grant no: 2019-00029). Funding by the Uppsala Diabetes Centre is gratefully acknowledged. Funding by The Swedish Science Council is gratefully acknowledged. Funding by the Olle Engqvist Byggmästare Foundation is gratefully acknowledged.

## Declaration of competing interest

Maria Strømme reports financial support was provided by The Swedish Science Council. Maria Strømme reports financial support was provided by Olle Engqvist Byggmästare Foundation. Jonas Lindh reports financial support was provided by Uppsala Diabetes Centre. Jonas Lindh reports financial support was provided by Sweden's Innovation Agency VINNOVA. The other authors declare that they have no known competing financial interests or personal relationships that could have appeared to influence the work reported in this paper.

## Data Availability

Data will be made available upon request.

## Appendix A Supplementary data

Supplementary material Image 1
