# Supplementary material for: Selective laser sintering of distinct drug and polymer layers as a novel manufacturing strategy for individually dosed tablets
Source: Int J Pharm X. 2025 May 21;9:100338. doi: 10.1016/j.ijpx.2025.100338 (PMC12152375; doi:10.1016/j.ijpx.2025.100338)
Supplement: Supplementary file 1 — Supplementary material [file mmc1.docx]

**Selective Laser Sintering of Distinct Drug and Polymer Layers as a Novel Manufacturing Strategy for Individually Dosed Tablets**

Jonas Autenrieth ^a^, Daniel Hedbom ^b^, Maria Strømme ^b^, Thomas Kipping ^c^, Jonas Lindh ^b^, Julian Quodbach ^d,‡^

^a^ Division of Molecular Pharmaceutics, Department of Pharmacy, Uppsala University, Uppsala Biomedical Center, P.O Box 580, SE-751 23 Uppsala, Sweden;

^b^ Division of Nanotechnology and Functional Materials, Department of Materials Science and Engineering, Uppsala University, Ångström Laboratory, Regementsvägen 1, Uppsala 751 03, Sweden

^c^ Merck Life Science KGaA, Frankfurter Str. 250, Postcode: D033/001, DE-642 93 Darmstadt, Germany;

^d^ Department of Pharmaceutics, Utrecht Institute for Pharmaceutical Sciences, Utrecht University, Universiteitsweg 99, 3584 CG Utrecht, Netherlands;

^‡^ Corresponding author. E-mail address: j.h.j.quodbach@uu.nl. Postal address: Universiteitsweg 99, Utrecht 3584 CG, Netherlands

Table of Contents

[1. Experimental setup 2](#_Toc197695483)

[2. UV-VIS absorption spectrum 3](#_Toc197695484)

[3. Fourier transform infrared spectroscopy (FTIR) 3](#_Toc197695485)

[4. Nuclear magnetic resonance (NMR) analysis 4](#_Toc197695486)

[5. Print temperature log 7](#_Toc197695487)

[6. Statistical analysis 9](#_Toc197695488)

[7. Thermogravimetric analysis (TGA) 9](#_Toc197695489)

[8. Experiments to find printing parameters 10](#_Toc197695490)

## Experimental setup


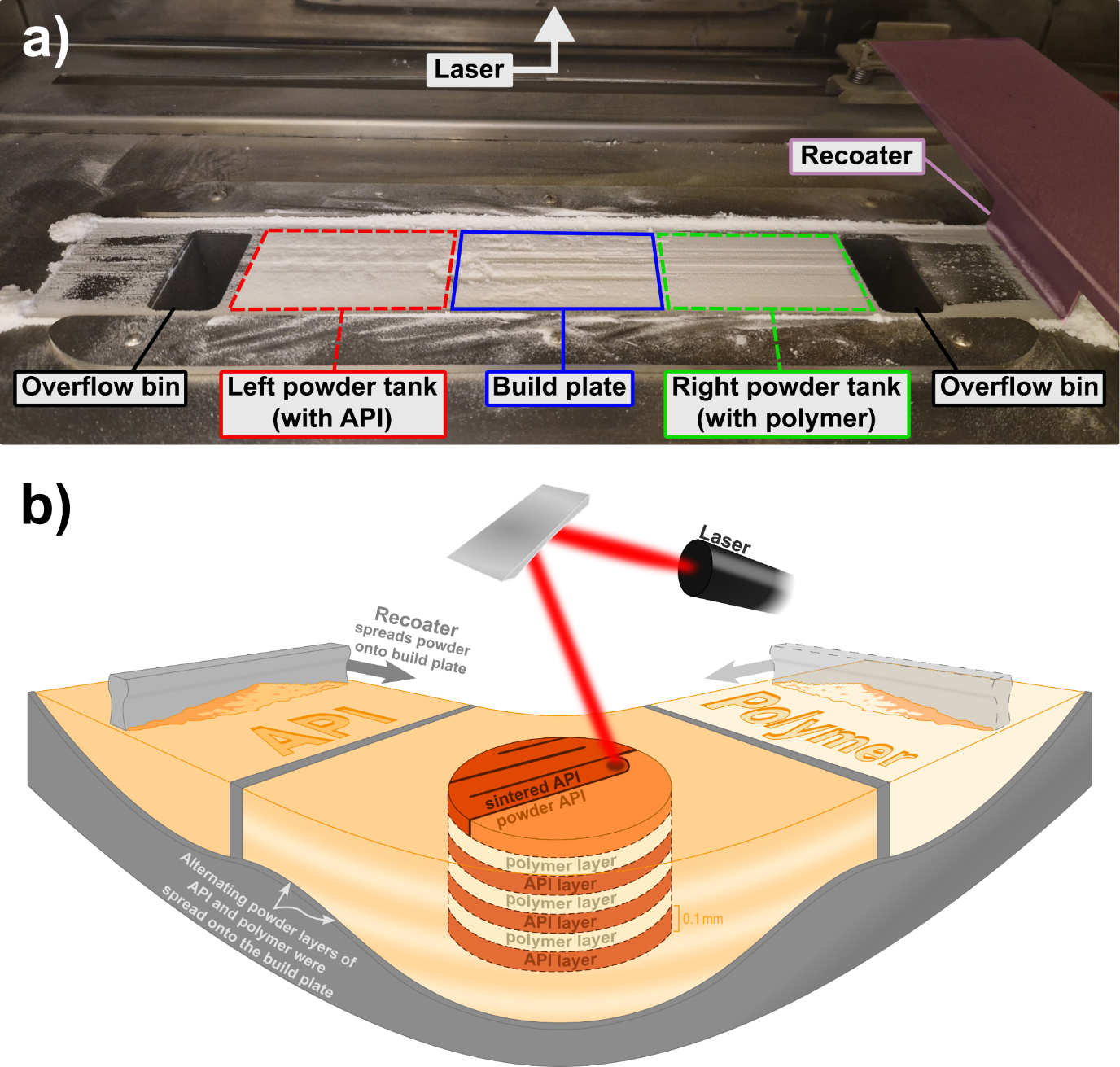


**Figure S 1.** Printing setup depicted as a) photo b) graphical visualization.

## UV-VIS absorption spectrum


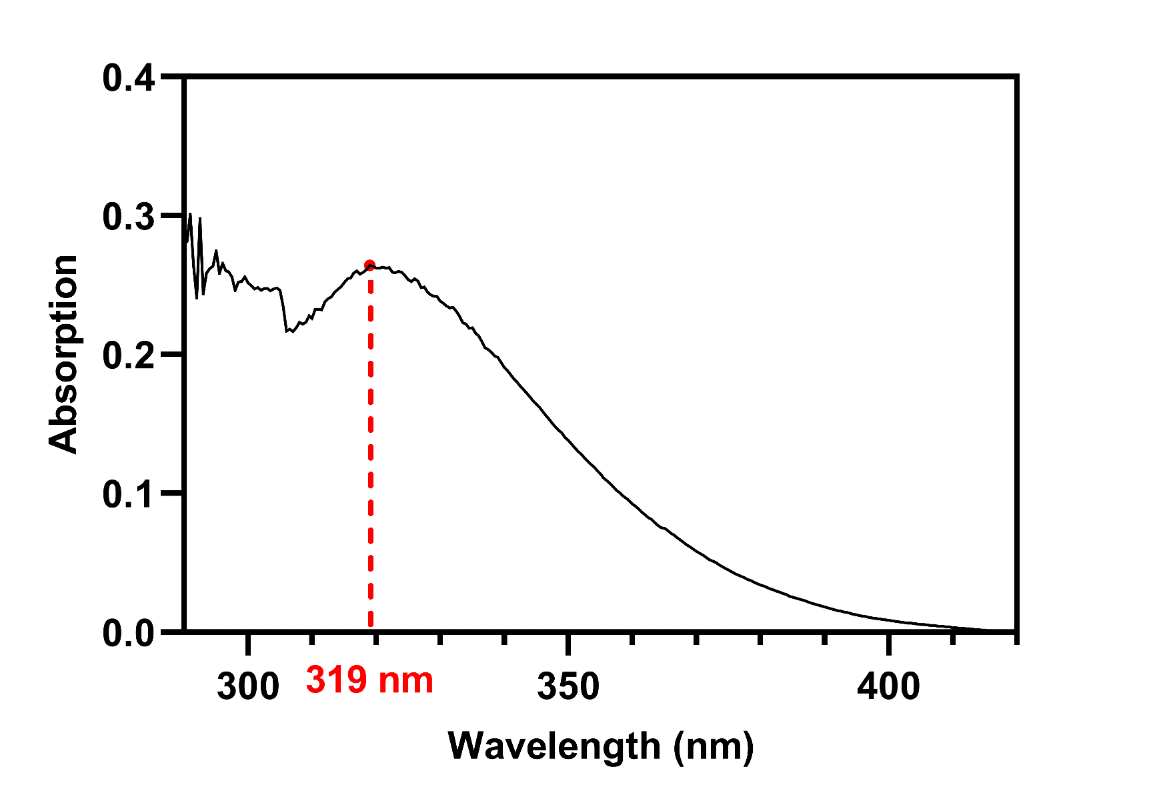


**Figure S 2.** UV-VIS spectrum of IND.

## Fourier transform infrared spectroscopy (FTIR)


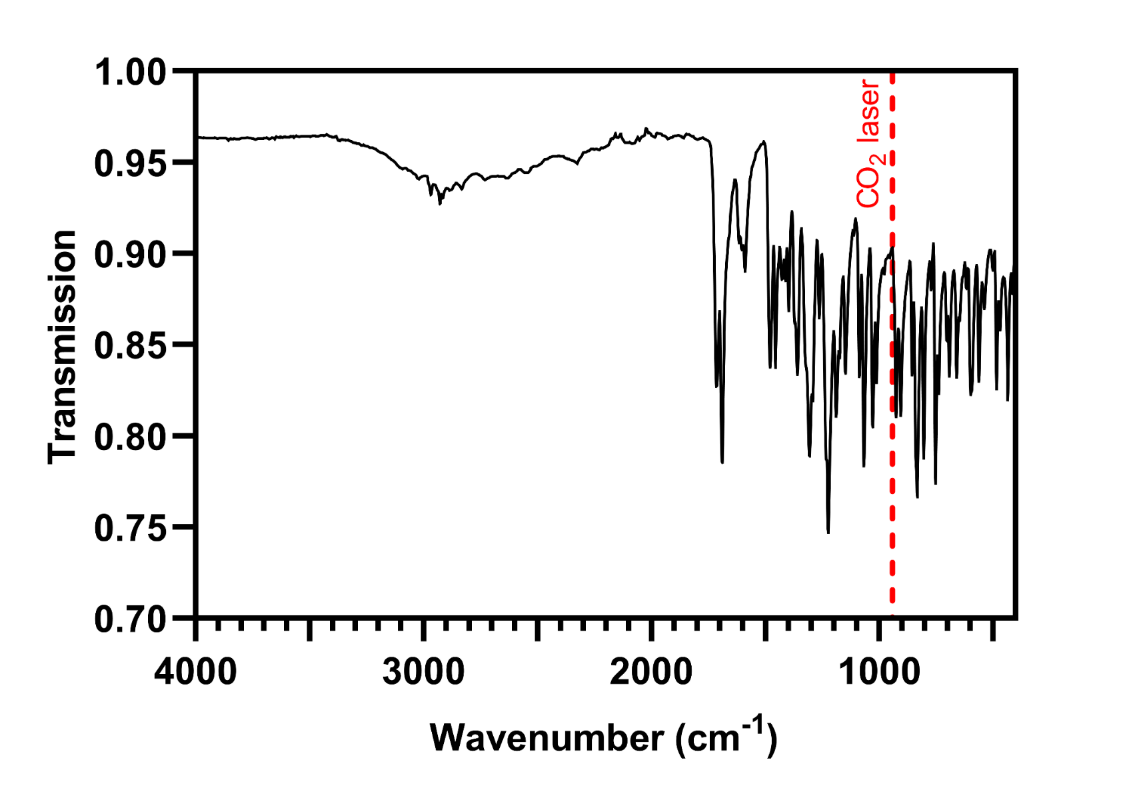


**Figure S 3.** FTIR analysis of IND powder shows absorbance at the same wavenumber as the laser emission.

## Nuclear magnetic resonance (NMR) analysis

**Figure S 4.1.** Indomethacin ^1^H-NMR (DMSO-d6): δ 12.36 (s, 1H), 7.71 – 7.61 (m, 4H), 7.04 (d, *J* = 2.6 Hz, 1H), 6.92 (d, *J* = 9.0 Hz, 1H), 6.72 (dd, *J* = 9.0, 2.6 Hz, 1H), 3.76 (s, 3H), 3.66 (s, 2H), 2.21 (s, 3H).

**Figure S 4.2.** ^1^H NMR (400 MHz, DMSO-d6): δ 4.73 – 4.15 (m, 1H), 3.83 (s, 1H), 1.64 – 1.22 (m, 2H).

**Figure S 4.3.** Stacked ^1^H NMR (400 MHz, DMSO-d6) spectra of printed tablet (type 4+1) and raw materials.

## Print temperature log


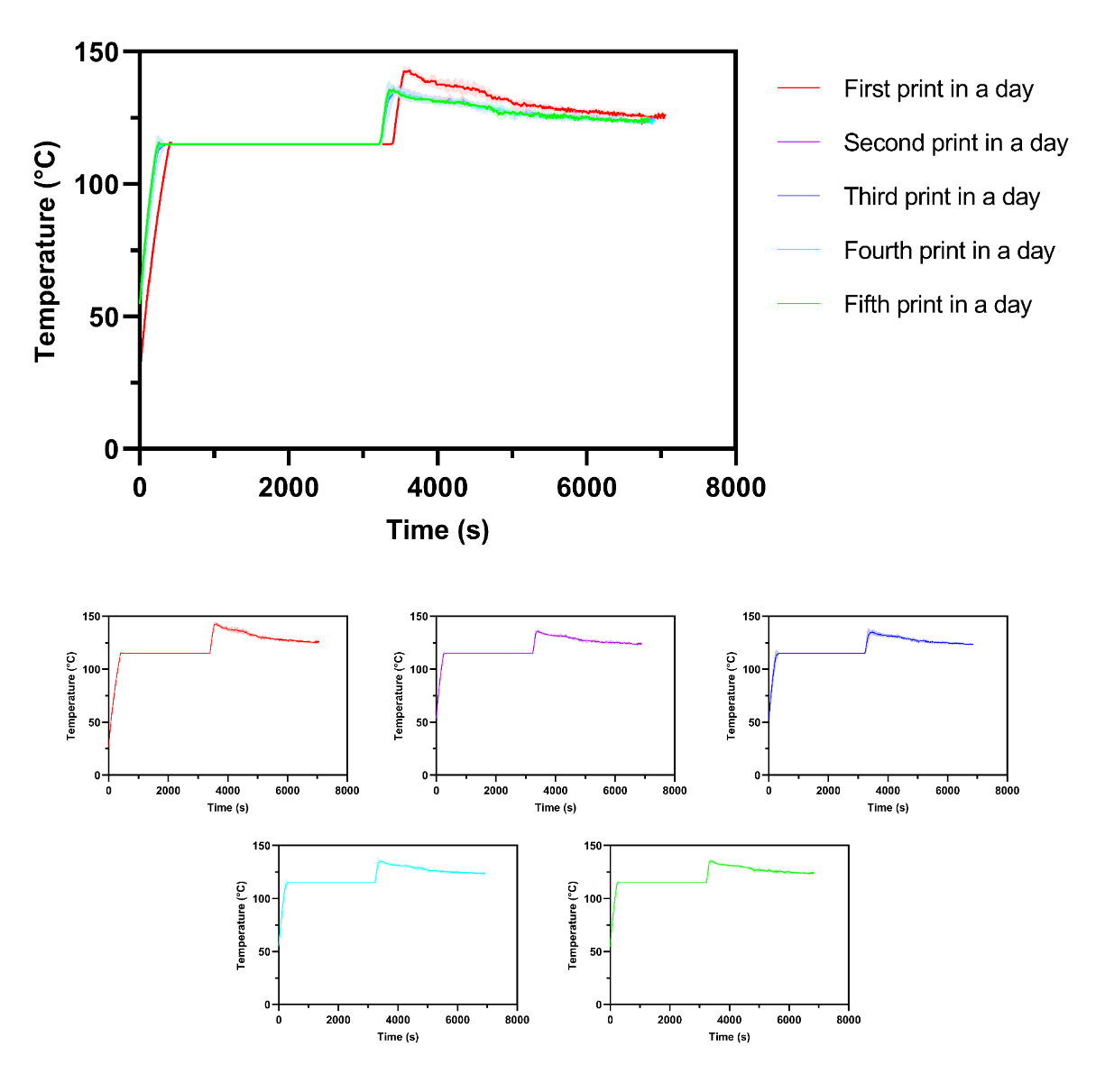


**Figure S 5.1.** Temperature of environmental air in the print chamber during a print. All temperature logs were averaged for each ordinal print number during the day and are depicted as average (line) with standard deviation (light colored area).


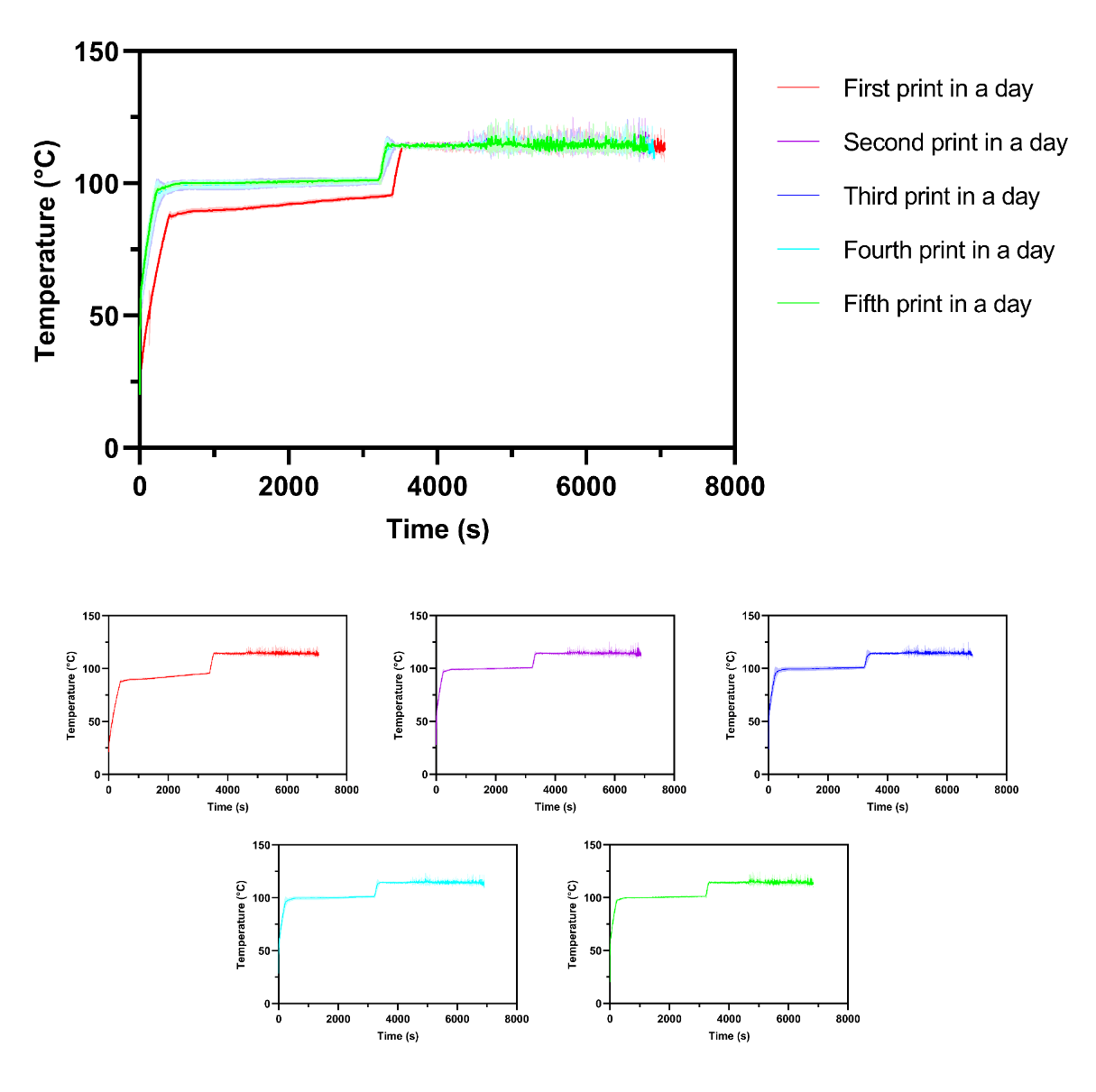


**Figure S 5.2.** Temperature of the powder bed during a print. All temperature logs were averaged for each ordinal print number during the day and are depicted as average (line) with standard deviation (light colored area).

## Statistical analysis

**Table S 1.** Sample sizes for statistical analysis of print success rates.

|  | Successful prints | Failed prints | Total prints |
| --- | --- | --- | --- |
| **Tablet type** |  |  |  |
| 3+1 | 5 | 4 | 9 |
| 4+1 | 5 | 3 | 8 |
| 5+1 | 6 | 3 | 9 |
| 6+1 | 6 | 4 | 10 |
| 8+1 | 7 | 6 | 13 |
|  |  |  |  |
| **Ordinal number throughout the day** |  |  |  |
| 1^st^ | 6 | 6 | 12 |
| 2^nd^ | 5 | 7 | 12 |
| 3^rd^ | 8 | 3 | 11 |
| 4^th^ | 7 | 3 | 10 |
| 5^th^ | 3 | 1 | 4 |
|  |  |  |  |
| **PVA type** |  |  |  |
| fresh | 20 | 17 | 37 |
| recycled | 9 | 3 | 12 |
|  |  |  |  |
| **All prints** | 29 | 20 | 49 |

## Thermogravimetric analysis (TGA)


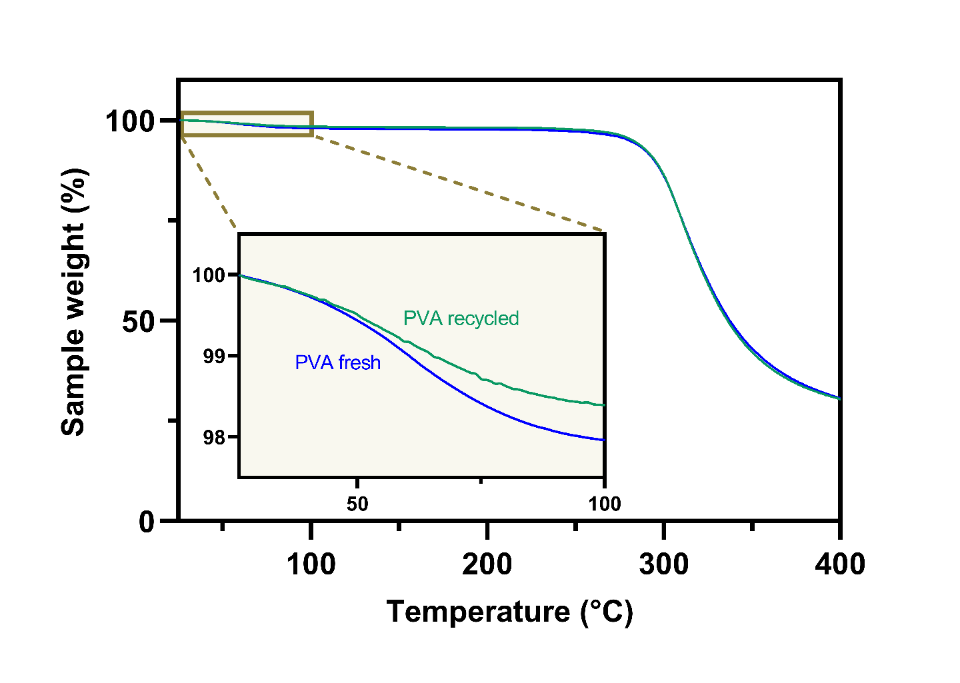


**Figure S 6.** TGA analysis of fresh and recycled PVA.

## Experiments to find printing parameters

**Table S 2.** Experiments conducted to find printing parameters, including scanning speed (SS), laser power (LP) and temperature (temp). Starting parameters were selected based on physicochemical properties, i.e., T_g_ and melting temperature of the polymers, in consideration of recommendations by the printer’s manufacturer. Initial tests were conducted with 3+1 and 5+1 tablets.

| **PVA parameters** | **IND parameters** | **Temp (°C)** | **Observation** | **Action taken** |
| --- | --- | --- | --- | --- |
| SS 60 000  LP 33 | SS 60 000  LP 33 | 95 | - failure in IND layers due to warping of all tablet types | - lower IND LP |
| SS 60 000  LP 33 | SS 60 000  LP 25 | 95 | - reduced warping, but low adhesion between PVA and IND leading to delamination | - lower IND SS |
| SS 60 000  LP 33 | SS 45 000  LP 25 | 95 | - high brittleness and delamination of lower layers | - increase temp |
| SS 60 000  LP 33 | SS 45 000  LP 25 | 100 | - reduced but still high brittleness | - increase temp |
| SS 60 000  LP 33 | SS 45 000  LP 25 | 105 | - less prints failed due to warping. Heat difference between irradiated and non-irradiated powder potentially too large. | - increase temp |
| SS 60 000  LP 33 | SS 45 000  LP 25 | 110 | - some warping observed. Potentially because of excessive laser power. | - lower PVA LP and IND LP |
| SS 60 000  LP 20 | SS 45 000  LP 20 | 110 | - successful prints, but tablets are very fragile | - increase PVA LP |
| SS 60 000  LP 23 | SS 45 000  LP 20 | 110 | - successful prints, but parts appear to be incompletely sintered | - increase IND LP |
| SS 60 000  LP 23 | SS 45 000  LP 23 | 110 | - warping observed | - increase temp |
| SS 60 000  LP 23 | SS 45 000  LP 23 | 115 | - successful prints, but still fragile | - increase PVA LP |
| SS 60 000  LP 26 | SS 45 000  LP 23 | 115 | - failure due to excessive warping of IND layers | - lower IND LP |
| SS 60 000  LP 26 | SS 45 000  LP 21 | 115 | - failure, delamination | - lower IND SS |
| SS 60 000  LP 26 | SS 42 000  LP 21 | 115 | - balling in IND layer and warping | - increase temp |
| SS 60 000  LP 26 | SS 42 000  LP 21 | 120 | - print fails early, IND powder on top of the supply tank melts and prevents powder spreading  - 115 °C determined as maximum temp due to melting of IND | - reset temp and IND SS, and lower IND LP |
| SS 60 000  LP 26 | SS 45 000  LP 19 | 115 | - multiple successful prints but some failures due to warping in IND layers | - increase IND SS |
| SS 60 000  LP 26 | SS 50 000  LP 19 | 115 | - good results for the tested 3+1 and 5+1 tablet types |  |
| expanding these parameters to other tablet types | | | | |
| SS 60 000  LP 26 | SS 50 000  LP 19 | 115 | - limited fails in 4+1 tablets due to warping | - lower PVA LP |
| SS 60 000  LP 25 | SS 50 000  LP 19 | 115 | - less fails in 4+1 tablets but still not fully acceptable | - repeat with 5+1 |
| SS 60 000  LP 25 | SS 50 000  LP 19 | 115 | - slightly more fails in 5+1 observed as compared to 4+1 tablets | - keep old settings |
| SS 60 000  LP 26 | SS 50 000  LP 19 | 115 | - 6+1 tablets were more fragile than 4+1 and 5+1 | - lower PVA SS |
| SS 55 000  LP 26 | SS 50 000  LP 19 | 115 | - 6+1 demonstrate acceptable mechanical strength |  |
| SS 60 000  LP 26 | SS 50 000  LP 19 | 115 | - 8+1 tablets were more fragile than 4+1, 5+1, and 6+1 | - increase PVA LP |
| SS 60 000  LP 27 | SS 50 000  LP 19 | 115 | - 8+1 tablets demonstrate acceptable mechanical strength | - increase PVA LP |
| SS 60 000  LP 28 | SS 50 000  LP 19 | 115 | - 8+1 tablets start to warp and fail more often |  |
| 🡪 use the previously found settings before exploring different tablet types for study | | | | |
